# The Powers of Participatory Medicine

**DOI:** 10.1371/journal.pbio.1001837

**Published:** 2014-04-15

**Authors:** Barbara Prainsack

**Affiliations:** Department of Social Science, Health & Medicine, King's College London, London, United Kingdom; Stanford University, United States of America

## Abstract

Does participatory medicine always empower patients, or could it also serve as a tool to shift responsibilities from other actors to individual patients?

This Perspective is part of the Public Engagement in Science series. The title of this article is inspired by Nikolas Rose's book *The Powers of Freedom: Reframing Political Thought* (Cambridge, UK: Cambridge University Press. 1999).

When the Californian personal genomics company 23andMe stopped disclosing health-related genetic risk information to new customers last December, a chapter in the history of personal genomics came to an end: 23andMe had been one of the last—and certainly the most prominent—companies still offering health-related genetic risk information direct-to-consumer. A few weeks earlier, the company had been ordered by the U.S. Food and Drug Administration (FDA) to halt operations until they could provide evidence of the validity of their claims to health benefits [1], and a class action lawsuit against the company had been filed: the plaintiffs alleged that the company was misleadingly advertising that their test results were useful for medical decision making [2].

Recent developments around 23andMe have inspired numerous critiques and commentaries in journals, blogs, and newspapers. Some authors have expressed their concerns about these developments and argued that the FDA overstepped their remit; others have applauded the effort to discipline a company that had gone too far: the main bone of contention was that 23andMe had been giving customers genetic risk information without clear evidence of clinical validity or clinical utility, and without involving customers' physicians. This, many commentators felt, had put their customers in danger.

But 23andMe had also disrupted things in a different sense, and this aspect has received little attention in the debate: 23andMe experimented with participatory medicine. Not only did the company give customers direct access to interpreted and raw genetic data on their genetic “risk” of being affected by a wide range of diseases, traits, and characteristics relevant to drug metabolism, but they also encouraged their customers to fill in surveys, upload information on phenotypes and lifestyle, and to suggest new research questions and projects. Compared to other genetic testing services, 23andMe was a more participatory endeavour—albeit one that has always also had clear commercial interests. The fact that the company, despite their strong rhetoric of participation and community, had filed for patents on discoveries made on the basis of their customers' data, for example, had led to a number of hiccups in previous months [3],[4].

This tension between the rhetoric of patient empowerment on the one hand, and the vested interests of powerful commercial actors on the other, is emblematic for the current situation of participatory medicine. Many of the references to participatory medicine in scholarly journals, mass media, and in the blogosphere at the moment use the language of participation to celebrate instances or visions of patient “empowerment.” Gilles Frydman and colleagues, for example, define participatory medicine as “a movement in which networked patients shift from being mere passengers to responsible drivers of their health, and in which providers encourage and value them as full partners” [5]. In this vision, the primary agency lies, however, still with traditional experts, who “encourage and value” patients as “full partners”; this means that for patients to be fully “empowered” they need to be encouraged and valued by others first. Also, the car metaphor is instructive: it is true that drivers are in charge of operating the car, but they are not always the ones who decide where to go. Moreover, they can only operate the car if they have enough money to get ahold of a car in the first place, and to pay for fuel.

A better picture of “genuine” participatory medicine—namely one that entails a genuine shift of power from traditional experts to patients and their families and friends—would include a system of roads that allows the people in different vehicles to go wherever they want to go; some vehicles are makeshift bikes while others are buses, or high-end sports cars. People can decide what vehicle to use, if any; they can decide whether they build it themselves, rent, or share it, and they are free in their choice of travel partners. The fact that people's freedoms are inevitably limited by the means and resources available to them merits a discussion in its own right. The key point here, however, is that genuine patient “empowerment” requires, first, the provision of publicly funded and regulated infrastructures that *de facto* enable choice, and that enable access to appropriate care (to stick with the car metaphor, the provision of a road system), and second, recognition that the decision *not* to drive a car is also an expression of a person's autonomy. The opportunity for participation must not become a duty. And while misdirected precaution often has unintended negative consequences [6]–[8], a certain level of regulation is necessary for participatory medicine to function.

It is *not* a requirement for genuine participatory medicine, however, that medical professionals “encourage” this participation. While many ethicists, clinicians, and regulatory agencies are still busy deliberating whether or not participatory medicine is worthy of their support, and discussing how patients can be protected in areas where they are seen as lacking sufficient competence or understanding, some people have already set up their own tools and infrastructures. The web-based platform CureTogether (curetogether.com), for example, was founded by two pioneers of the self-tracking movement, Alexandra Carmichael and Daniel Reda, in 2008. On this platform, users quantify and share information about the nature and severity of symptoms, as well as treatment responses. The site then aggregates and analyses (anonymised) data so that users can see what treatments work for people with similar symptoms, comorbidities, or demographic parameters [9]. The platform was acquired by 23andMe in 2012 with the idea that people would upload their genome data to the CureTogether platform and use these as additional parameters to find patients who are similar to them. Given that 23andMe is now in the process of redefining its business model and mission, this plan is unlikely to go ahead. What this example illustrates, however, is that if public actors in the health domain ignore citizen-led platforms because they view them as elitist or ethically problematic (or both), commercial companies may acquire them [10]. The failure of public actors to provide support to, or take co-ownership of, citizen-led initiatives is a missed opportunity to ensure that they are run in a responsible and accountable manner.

What also becomes clear from these examples is that there is not *one* participatory medicine that universally empowers patients. While participation often does increase the agency of people in meaningful ways, it could also serve as an excuse to devolve responsibilities and costs to individuals. The “big society” vision of some political parties is an example: citizens who help themselves and others because national and local governments have stopped providing infrastructures are applauded for being active participants in society, while those who are not strong enough to help themselves can be dismissed as unwilling. Moreover, it is possible that patients make contributions—in terms of time, data, or information—that other entities will profit from, without receiving any share in these profits, also under the label of “participatory medicine” or patient “empowerment”; this is what led to the “patent crisis” around 23andMe [3],[4]. Thus, we need to take a close look at how power and agency are distributed in a specific context or account of participatory medicine: Who decides what will be done? Who will benefit from the process, and in what ways? Can people opt out, and if so, at what costs [11],[12]? What research ethics requirements should be met when studies or trials are carried out outside of traditional infrastructures, especially when they are led by patients [13]? With regard to web-based tools used in participatory medicine platforms specifically, the need to address new challenges pertaining to data protection is also particularly pressing [14]–[16].

There is a growing number of truly exciting initiatives that have already started to make medicine more participatory, in the deep meaning of the word: they increase the space within which patients and their families and friends can make decisions that are meaningful to them, embedded in infrastructures that are socially just and represent the broadest of interests. They do this by improving medical literacy, by enabling patients to engage with data and information that are relevant to them, to connect with others who may have relevant experience or expertise, or by facilitating mutual support. They refrain from prejudicing what kinds of data, information, or practices will be useful to patients, but they allow people to decide for themselves (with the help of others, if they so desire). And they are very transparent about their commercial stakes and interests, as well as about how data obtained from patients will be stored, processed, and utilised [11]. Such initiatives deserve the support of public actors; it is not sufficient to assume that the powers of “the web” will facilitate true participatory medicine. Such support would also mean that funding programmes for research and innovation channel resources into initiatives fostering and facilitating participation.

## References

[1] FDA (2013) 23andMe, Inc. 11/22/13. Available: http://www.fda.gov/ICECI/EnforcementActions/WarningLetters/2013/ucm376296.htm. Accessed 17 February 2014.

[2] Conley J (5 December 2013) The revolt of the Cs: class action filed against 23andMe. Genomics Law Report. Available: http://www.genomicslawreport.com/index.php/2013/12/05/the-revolt-of-the-cs-class-action-filed-against-23-and-me/. Accessed 17 February 2014.

[3] Sterckx S, Cockbain J, Howard HC, Borry P (2013) “I prefer a child with…”: designer babies, another controversial patent in the arena of direct-to-consumer genomics. Genet Med 15: 923–924. 24091802 10.1038/gim.2013.164

[4] Prainsack B (8 October 2013) 23andMe's “designer baby” patent: when corporate governance and open science collide. Genomes Unzipped Available: http://www.genomesunzipped.org/2013/10/guest-post-23andmes-designer-baby-patent-when-corporate-governance-and-open-science-collide.php. Accessed 17 February 2014.

[5] Frydman G (2013) A patient-centric definition of participatory medicine. Available: http://participatorymedicine.org/epatients/2010/04/a-patient-centric-definition-of-participatory-medicine.html. Accessed 17 February 2014.

[6] Prainsack B, Reardon J, Hindmarsh R, Gottweis H, Naue U, et al. (2008) Misdirected precaution? Nature 456: 34–35. 18987720 10.1038/456034a

[7] Green RC, Farahany NA (2014) The FDA is overcautious on consumer genomics. Nature 505: 286–287. 24436984 10.1038/505286a

[8] Harford T (14 February 2014) The murkier side of transparency. The Financial Times. Available: http://www.ft.com/cms/s/2/785bd614-9378-11e3-b07c-00144feab7de.html#axzz2tUYPvcYY. Accessed 17 February 2014.

[9] Prainsack B (2013) Let's get real about virtual: online health is here to stay. Genet Res (Camb) 95: 111–113. 23902852 10.1017/S001667231300013X

[10] Prainsack B (2011) Voting with their mice: personal genome testing and the “participatory turn” in disease research. Account Res 18: 132–147. 21574070 10.1080/08989621.2011.575032

[11] Prainsack B (2014) Understanding participation: the ‘citizen science’ of genetics. In: Prainsack B, Schicktanz S, Werner-Felmayer G, editors. Genetics as social practice. Farnham: Ashgate. pp. 147–164..

[12] Bucci M, Neresini F (2007) Science and public participation. In: Hackett EJ, Amsterdamska O, Lynch M, and Wajcman J, editors. The handbook of science and technology studies. Cambridge, MA: The MIT Press. pp. 448–472.

[13] Vayena E, Tasiouslas J (2013) The ethics of participant-led biomedical research. Nat Biotechnol 31: 786–787. 24022150 10.1038/nbt.2692

[14] Kang J, Shilton K, Estrin D, Burke J, Hansen M (2012) Self-surveillance privacy. Iowa Law Rev 97: 809–847.

[15] Peppet SR (2012) Privacy & the personal prospectus: should we introduce privacy agents or regulate privacy intermediaries? Iowa Law Review Bulletin 97: 77–93.

[16] Watson SM (12 November 2013) You are your data. Slate. Available: http://www.slate.com/articles/technology/future_tense/2013/11/quantified_self_self_tracking_data_we_need_a_right_to_use_it.html. Accessed 17 February 2014.

